# Supplementary material for: Non-perturbative cathodoluminescence microscopy of beam-sensitive materials
Source: Nanophotonics. 2025 Mar 6;14(11):2095–101. doi: 10.1515/nanoph-2024-0724 (PMC12133309; doi:10.1515/nanoph-2024-0724)
Supplement: Supplementary file 1 — Supplementary Material Details [file j_nanoph-2024-0724_suppl_001.pdf]

Research Article

Malcolm Bogroff*, Gabriel Cowley*, Ariel Nicastro*, David Levy, Yueh-Chun Wu, Nannan Mao, Tilo H. Yang, Tianyi Zhang, Jing Kong, Rama Vasudevan, Kyle P. Kelley, and Benjamin J. Lawrie

Supporting Information

Non-perturbative cathodoluminescence microscopy of beam-sensitive materials

S1 Analysis of CL spectrum image in Figure 1

Figure S1 provides a framework for understanding the CL spectrum images shown in the manuscript. Figure S1(a) reproduces the SEM image shown in Fig. 1(a) of the manuscript for reference, and Fig. S1(b) illustrates the calculated explained variance of the NMF decomposition shown in Fig. 1(c) and (d) of the manuscript for NMF decompositions of 1-6 components (with explained variance values of $\{0.40, 0.75, 0.82, 0.85, 0.8, 0.88\}$ for $N = \{1, 2, 3, 4, 5, 6\}$. We chose to use 3 NMF components throughout the manuscript based on a qualitative judgement that the explained variance of 0.82 was sufficient and because two components were sufficient to qualitative separate the hBN luminescence from the substrate luminescence, but a third component highlighted the heterogeneity of the narrowband color center luminescence within the hBN flake as shown in the manuscript. Additional components provided no additional physically meaningful information.

The explained variance shown in Fig. S1(b) was limited by electron-beam modification of defect bands that occurred during these measurements and by the slight variation in CL spectra as a function of position across the flake. In order to provide further context for the NMF reconstruction shown in the manuscript, Fig. S1(c) and (d) illustrate the raw intensity map of the measured CL (summed over wavelengths of 523-543 nm) and point spectra acquired at three points on the sample. The substrate itself exhibits broadband luminescence that is well known in the literature as shown in the pink spectrum in Fig. S1(d) for the point highlighted in Fig. S1(c). The hBN defect luminescence is observed across the hBN flake as a result of a high defect density in this flake, but the intensity of the observed defect band varies substantially as a function of position, as shown by the red and black spectra acquired at the red and black points shown in Fig. S1(c).

S2 Dose dependent defect modification in hBN

The top element of Fig. S2 illustrates the measured counts at wavelengths of 417 nm, 533 nm, and 647 nm extracted from Fig. 1(b) in the manuscript. Notably, the 647 nm band associated with substrate luminescence is largely insensitive to the electron beam while the 533 nm line associated with hBN color centers is bleached by the electron-beam exposure within $\sim 8s$, corresponding to an electron-beam dose of $\sim 3.5 \times 10^5 \mu C/cm^2$. An additional broad defect band near 417 nm is initially absent from the CL spectra, but it grows substantially in intensity with increasing exposure time.

When the beam energy increases to 10 kV (as illustrated by the time series traces in the bottom of Fig. S2), the 533 nm color center luminescence is completely bleached in less than 1s, corresponding to an electron-beam dose of $\sim 4.7 \times 10^4 \mu C/cm^2$, and similar rapid, but temporary, bleaching of the substrate defect band is observed. The 417 nm defect band that was created under 5kV excitation is not observed under 10kV excitation.

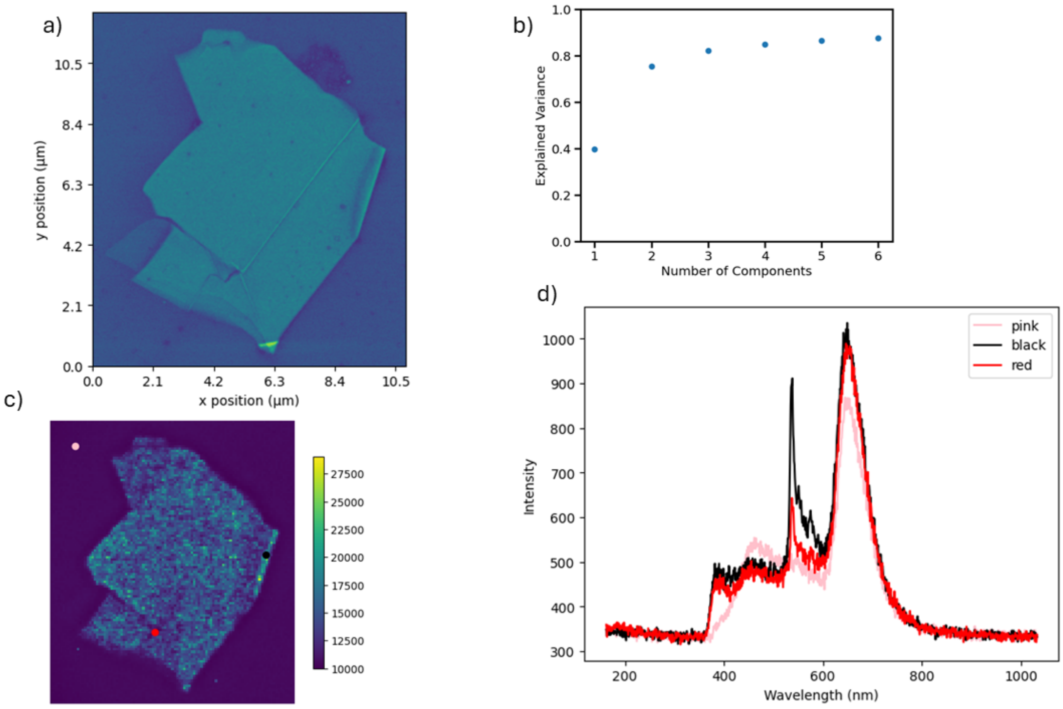

Fig. S1: a) SEM image of sample. b) Explained variance of NMF reconstruction shown in Fig. 1 of the manuscript c) Intensity map of raw CL summed over wavelengths of 523-543 nm. d) Point spectra at 3 points of interest acquired at the points marked by the color-coded points in (c).

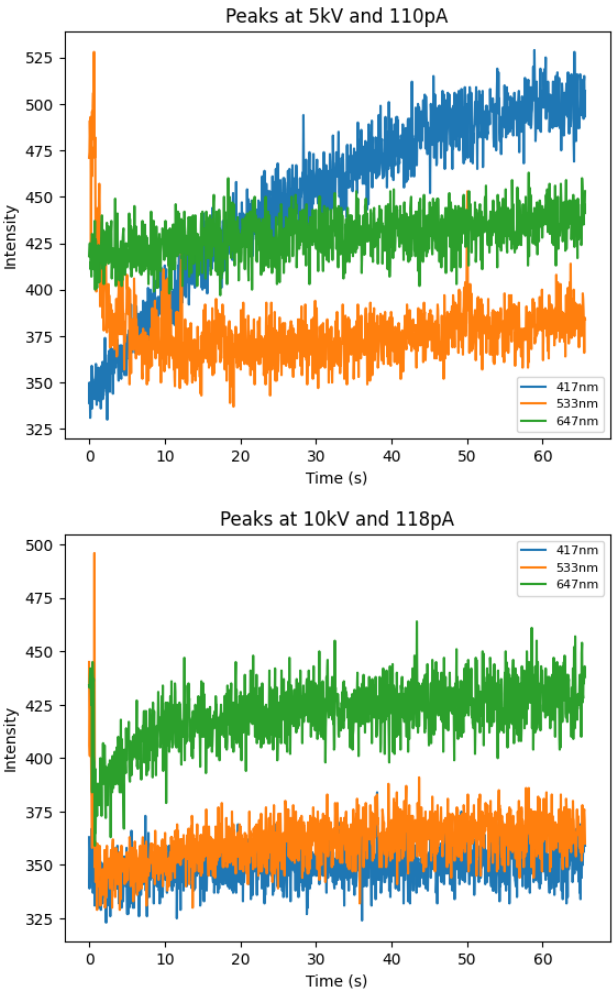

Fig. S2: Evolution of emission at 3 wavelengths of interest (417 nm, 533 nm, and 647 nm) under electron-beam excitation at 5 kV and 10 kV.
